# Scale dependence of sex ratio in wild plant populations: implications for social selection

**DOI:** 10.1002/ece3.1958

**Published:** 2016-02-03

**Authors:** Brian J. Sanderson, Malcolm E. Augat, Douglas R. Taylor, Edmund D. Brodie

**Affiliations:** ^1^ Mountain Lake Biological Station and Department of BiologyUniversity of Virginia485 McCormick RoadCharlottesvilleVirginia22904

**Keywords:** Context‐dependence, gynodioecy, local mate competition, multilevel selection, population structure, *Silene vulgaris*

## Abstract

Social context refers to the composition of an individual's social interactants, including potential mates. In spatially structured populations, social context can vary among individuals within populations, generating the opportunity for social selection to drive differences in fitness functions among individuals at a fine spatial scale. In sexually polymorphic plants, the local sex ratio varies at a fine scale and thus has the potential to generate this opportunity. We measured the spatial distribution of two wild populations of the gynodioecious plant *Silene vulgaris* and show that there is fine‐scale heterogeneity in the local distribution of the sexes within these populations. We demonstrate that the largest variance in sex ratio is among nearest neighbors. This variance is greatly reduced as the spatial scale of social interactions increases. These patterns suggest the sex of neighbors has the potential to generate fine‐scale differences in selection differentials among individuals. One of the most important determinants of social interactions in plants is the behavior of pollinators. These results suggest that the potential for selection arising from sex ratio will be greatest when pollen is shared among nearest neighbors. Future studies incorporating the movement of pollinators may reveal whether and how this fine‐scale variance in sex ratio affects the fitness of individuals in these populations.

## Introduction

The fitness consequences of interacting phenotypes – traits involved in social or sexual interactions – depend on the context in which these interactions occur (Moore et al. 1997). Social context is a ubiquitous feature of natural populations and can be created by demography, the spatial structuring of populations, and the patterns of interactions among individuals within populations. Social selection is a form of multilevel selection that acts on, and results from, the phenotypes of all interacting individuals in a population (Crook 1972; West‐Eberhard 1979; Wolf et al. 1999). Thus, the phenotypes of individuals and their social partners act as both the targets and agents of selection, generating complex evolutionary dynamics across levels of biological organization (Moore et al. 1997). Social selection drives the evolution of combat traits in forked fungus beetles (Formica et al. 2011, 2012), intrasexual aggressive behavior in water striders (Eldakar et al. 2010), and reproductive conflicts in the body size of worker ants (Tsuji 1995). In each of these cases, the magnitude and direction of selection on the trait of interest is in part determined by the social context in which the trait is expressed.

We know little about the spatial scale on which social selection is likely to act in plants or animals. Social selection has largely been studied as effects on individuals arising from the average phenotype expressed in a population (e.g., the average body size of males; Formica et al. 2011), yet different social contexts can exist and vary across space and time at a fine scale within populations. Under these circumstances, individuals experience continuously varying, and potentially overlapping, social contexts across the population, and so understanding the scale on which organisms interact within populations is critical to understanding the potential fitness consequences of social interactions.

Wolf et al. (1999) presented a general model of social selection demonstrating how a covariance between the phenotypes of social partners affects selection on interacting phenotypes. The total selection differential on the trait of interest in this model is described by:

$$S_{i}=P_{ii}\beta_{N}+C^{ij{}^{\prime }}\beta_{S}$$

where *P*_*ii*_ is the phenotypic variance of the trait, *β*_*N*_ is the nonsocial or natural selection gradient acting directly on the trait of the individual, *C* ^*ij*′^ is the covariance among individual trait values and the phenotypes of their social partners, and *β*_*S*_ is the social selection gradient representing the relationship between individual fitness and the trait values of social partners. The product *P*_*ii*_ *β*_*N*_ is the nonsocial selection differential, representing selection directly on the trait of interest. The social selection differential *C* ^*ij*′^ *β*_*S*_ represents selection arising from the phenotypes of social partners. The critical metric that determines whether there is potential for social selection is this covariance between partners, or neighbors, *C* ^*ij*′^. When *C* ^*ij*′^ is nonzero, the total selection differential is the sum of the nonsocial and social selection differentials. However, when no consistent pattern of phenotypic covariance exists (*C* ^*ij*′^ = 0), the traits of social partners (*j* ^′^ do not exert net selection on traits in the focal individuals, and only direct selection on the individual will affect phenotypic evolution.

The spatial scale of interaction is likely to be a primary determinant of *C* ^*ij*′^, especially in nonmotile organism such as plants, where fitness impacting interactions are determined by physical proximity or the movement of animal vectors. Plants compete for resources in the soil and to avoid shading (Bengtsson et al. 1994; Dykstra et al. 2009; Van Etten and Chang 2009; Weiner and Freckleton 2010), and stand‐level characteristics can result in both competition and facilitation for pollinators, in addition to affecting the apparency of the stand to herbivores (Stevens et al. 1995; Kelly 1996; Aspi et al. 2003; Donohue 2004; Bartkowska and Johnston 2014). Considerable attention has been paid to the degree to which the fitness effects of population‐ and community‐level phenotypes depend on the spatial scale of interactions (Barrett and Thomson 1982; House 1992; Aizen 1997; Stehlik et al. 2008; Lin et al. 2015). If all individuals experience similar social contexts, as when stand‐level patterns influence fitness, then we expect no opportunity for social selection. As the scale of interaction reduces, as expected under local competition with neighboring individuals or short range movements of pollinators, we expect greater opportunity to arise as individual plants experience variable contexts. The importance of spatial scale may be more severe in plants than in animals because the ability of plants to respond to selective interference through behavioral modification is likely limited to shifts in phenology (e.g., Rivkin et al. 2015; Weis et al. 2015).

In this study, we focus on fitness effects arising from the sex of neighboring plants across various spatial scales using social selection analysis. Sex ratio is a social characteristic of populations or neighborhoods that is often linked with individual fitness because the frequency of available mates is defined by the demographic composition of the population (Fisher 1930; Hamilton 1967). In particular, in plant populations, the spatial structure of the sexes generates a high degree of fine‐scale variance in the sex ratio experienced by individuals (McCauley and Taylor 1997). Fine‐scale variance in sex ratio is likely to result in strong fitness consequences because the fitness of individuals in a population will depend not only on their own sex allocation and behavior, but also on the availability of compatible mates and the strength of competition with neighboring individuals (Hamilton 1967).

In this article, we measure the spatial distribution of individuals within wild plant populations to examine the variation in local neighborhood sex ratios at varying scales. We address the following questions: (1) How do patterns of phenotypic covariance of sex vary among spatial scales; and (2) What do these patterns suggest about the opportunity for social selection in these populations? We demonstrate that the greatest variance in sex ratio among individuals occurs at the smallest spatial scales and that the greatest covariance between the sex of focal individuals and the local sex ratio also occurs at the smallest spatial scales. These patterns suggest that the scale of mating will determine the degree to which selection differentials differ among individuals within the population. Selection differentials will be most strongly impacted by social interactions when pollen is shared among nearest neighbors, and dissipate if mating occurs over larger spatial scales. We discuss these results in terms of their implications for social selection arising from sex ratio and highlight the generality and utility of the social selection framework for the study of plant mating systems and pollination biology.

## Materials and Methods

### Study system

*Silene vulgaris* (Caryophyllaceae) is a gynodioecious perennial plant that grows predominantly along roadsides in its introduced range in North America (Taylor and Keller 2007). There is tremendous variation in sex ratio among populations, ranging between 30% and 100% hermaphrodite (McCauley et al. 2000). As in many gynodioecious species, sex is determined through cytonuclear interactions: mutations in the mitochondrial genome cause the abortion of anthers, resulting in functionally female plants, while nuclear‐encoded proteins can repress the action of the mitochondrial mutations, resulting in hermaphroditic plants (Frank 1989; Charlesworth and Laporte 1998). The species is pollinated both by diurnal (syrphid flies and bumble bees) and nocturnal insects (noctuid moths; Marsden‐Jones and Turril 1957; Stone 2013). Seeds are passively dispersed when fruits dehisce, which may explain the high degree of genetic structuring of populations (Olson et al. 2006).

### Mapping of populations

We sampled plants in a large, approximately 10 hectare pasture in Simmonsville, VA, wherein *Silene vulgaris* is patchily distributed. In October 2012, we ran 100 meter transects across the entire pasture and selected two large patches of very high density for fine‐scale mapping. Each plant was assigned a unique ID, and its sex was determined by the presence of mature anthers (hermaphrodite) versus aborted anthers (female). We mapped the spatial positions for all individuals in the two populations through trilateration: Three tripods were spaced evenly around the perimeter of the population, and the GPS coordinates of these tripods were recorded using the Trimble TSC1 survey platform (Trimble Navigation, Sunnyvale, CA). The measurement of the GPS coordinates for each of these reference points was estimated as accurate to below a distance of one meter. The distance from each plant to each of these three tripods was recorded using the Sonin Multi‐Measure Combo Pro sonic range finder (Sonin Inc., Charlotte, NC). We used a custom Python trilateration script to convert each of these ordered triplets of distances into GPS coordinates for each individual plant (script available at Dryad http://dx.doi.org/10.5061/dryad.c06n3). All of the following analyses were performed using R version 3.1.1 (R Core Team 2014).

### Quantifying social context

We used the function dist to create matrices of the pairwise distance (in meters) between all plants for each subpopulation. We then quantified local hermaphrodite frequencies for each individual by tallying the number and sex of neighboring individuals at neighborhood sizes ranging from 0.5 to 6.0 m radii (in 0.5 m steps) from the focal plant. Throughout the following analyses, the hermaphrodite frequency does not include the focal individual, which is the convention in social selection analysis (Wolf et al. 1999). We plotted these spatial relationships using the function ggplot in the package ggplot2 (Wickham 2009).

### Variance in hermaphrodite frequency at different spatial scales

To determine whether the hermaphrodite frequency experienced by individuals significantly varied among spatial scales, we used the function leveneTest in the package car, which includes an implementation of the Brown–Forsythe test for heterogeneity of variances (Fox and Weisberg 2011). We tested whether mean hermaphrodite frequency experienced by individuals differed among spatial scales with a generalized linear mixed model with a binomial family error distribution and logit link using the function glmer in the package lme4 (Bates et al. 2014). In this model, we regressed hermaphrodite frequency on sex, radius of spatial scale, the sex‐by‐spatial‐scale interaction as fixed effects, and plant ID as a random effect. Although there was significant heterogeneity of variances in hermaphrodite frequency among spatial scales, this did not appear to have a strong effect on the fit of the mixed model (based on a visual inspection of the residuals and predicted values of the model) and so we did not adjust the model to account for heteroscedasticity. To determine the significance of these parameter estimates, we used the function Anova in the package car, specifying type‐III sums of squares (Fox and Weisberg 2011).

We calculated the phenotypic covariance among social partners (*C* ^*ij*′^) and the phenotypic variance of local hermaphrodite frequencies (*P*_*jj*_) across spatial scales using the functions cov and var. We tested whether the *C* ^*ij*′^ terms were significantly different from zero using the function cor.test. Finally, we calculated the correlation of the local hermaphrodite frequencies experienced across the different spatial scales and tested the significance of these correlations with the function corr.test in the package psych (Revelle 2015). Whenever a population is comprised of continuously overlapping social neighborhoods, the social phenotypes experienced by neighboring individuals will include some of the same individuals and thus violate assumptions of statistical independence. Some researchers, such as Bartkowska and Johnston (2014), have used permutation tests of nonoverlapping social neighborhoods to estimate the degree to which violations of model assumptions are driving spurious results. This approach is less helpful when the patterns of phenotypic correlations are scale‐dependent, and the potential scales of interaction are large relative to the population as a whole, so caution should be used in interpreting the significance of results.

The spatial data, Python trilateration script, and the R scripts described in these methods are available at Dryad http://dx.doi.org/10.5061/dryad.c06n3.

## Results

We mapped 278 plants in the first population (“Burk1”) and 390 plants in the second population (“Burk2”). The population‐level hermaphrodite frequency of Burk1 was 0.658, and the population‐level hermaphrodite frequency of Burk2 was 0.590. However, there was significant heterogeneity in the distribution of the sexes within each population (Table 1).

**Table 1:** Mean and variance of sex ratio and number of social partners experienced by individuals across spatial scales measured as radii from 0.5 m to 6.0 m

Scale (m)	Mean hermaphrodite frequency	Variance in hermaphrodite frequency	Mean number of neighbors
0.5	0.630	0.174	2.0
1.0	0.611	0.080	5.8
1.5	0.622	0.043	11.9
2.0	0.621	0.027	20.0
2.5	0.625	0.018	30.0
3.0	0.621	0.014	41.5
3.5	0.621	0.011	54.2
4.0	0.618	0.009	68.5
4.5	0.615	0.007	83.8
5.0	0.613	0.006	99.3
5.5	0.612	0.006	116.1
6.0	0.611	0.005	133.1

**Figure 1:**
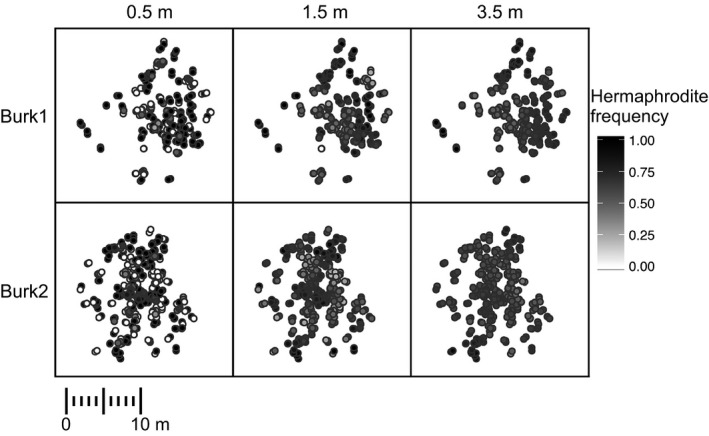
Locally experienced hermaphrodite frequency for all individuals in two wild populations at different spatial scales. Each point represents an individual, and the shading of the point represents the subjective local frequency of hermaphrodites for that individual assessed at radii from 0.5, 1.5, and 3.5 m. Lighter shading represents individual plants that experienced a low frequency of hermaphrodites, darker shading represents individuals that experienced a high frequency of hermaphrodites. The *X*‐axis and *Y*‐axis are arbitrary Euclidian distance in m, with the scale as indicated by the scale bar.

**Figure 2:**
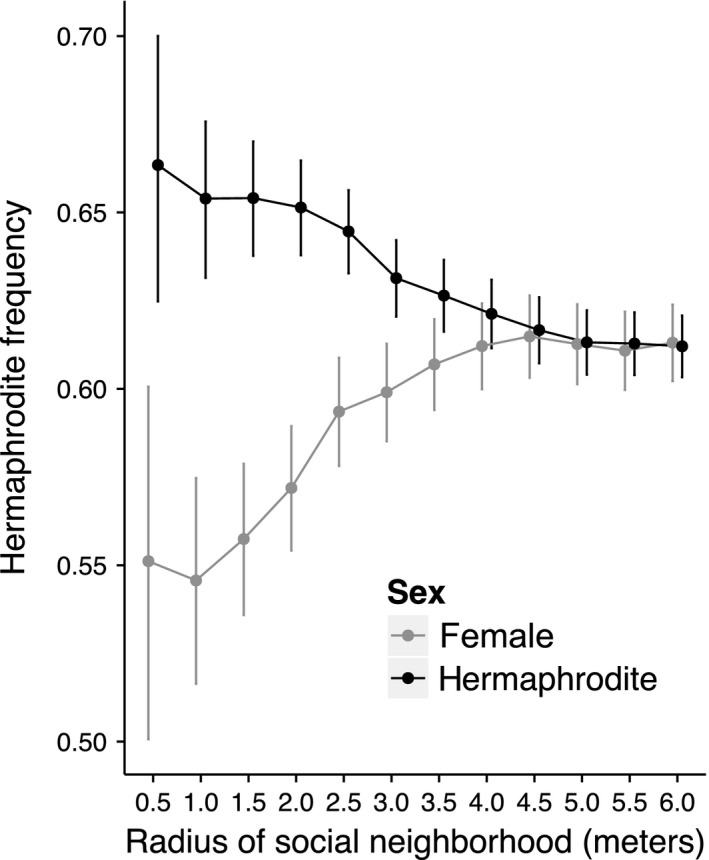
Hermaphrodite frequency experienced by hermaphrodites (black) and females (gray) across spatial scales measured in radii from 0.5 m to 6.0 m. The points are least square means of hermaphrodite frequency regressed on sex, radius of spatial scale, the sex‐by‐scale interaction as fixed effects, and plant ID as a random effect. Lines represent 95% confidence intervals of the LS means.

The mean hermaphrodite frequency of all individuals varied significantly among spatial scales (Table 2). There was a significant interaction of sex and spatial scale; hermaphrodites experienced a larger frequency of hermaphrodites among their neighbors, while females experienced a smaller frequency of hermaphrodites (Table 2). As the size of the spatial neighborhood increased, the hermaphrodite frequency experienced by both hermaphrodites and females converged on the population‐level hermaphrodite frequency (Table 2; Figs. 1, 2).

**Table 2:** Type‐III sum of squares test of significance for generalized linear mixed model of hermaphrodite frequency on sex, spatial‐scale, and the sex‐by‐scale interaction

Parameter	*χ* ^2^	df	*P*‐value
Sex	12.39	1	<0.001
Spatial scale	95.70	11	<0.001
Sex × scale	205.27	11	<0.001

**Figure 3:**
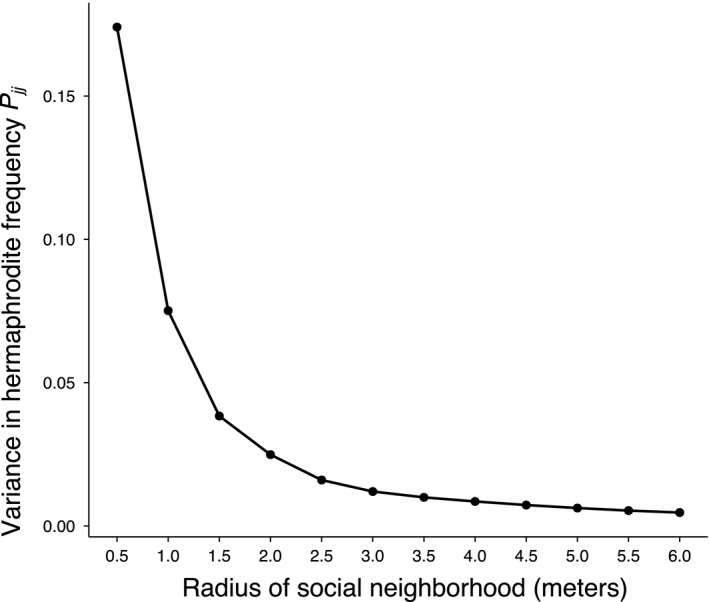
Variation in local hermaphrodite frequency decreases continuously with increasing spatial scale. Each point represents the variance in local hermaphrodite frequency assessed at spatial scales along the *X*‐axis from 0.5 m to 6.0 m in 0.5 m steps.

**Figure 4:**
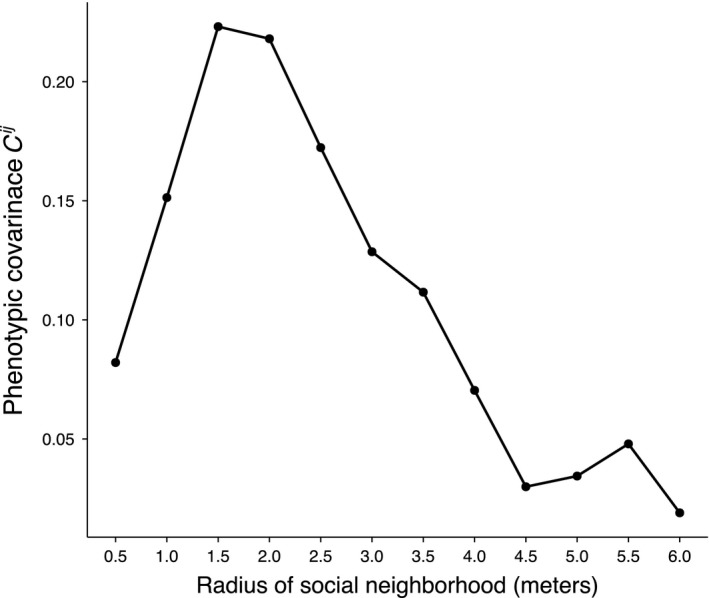
Phenotypic covariance decreases with increasing spatial scale. Each point represents the variance‐standardized covariance between the sex of individuals and the local frequency of hermaphrodites assessed at spatial scales along the *X*‐axis from 0.5 m to 6.0 m in 0.5 m steps.

The variance in hermaphrodite frequency greatly differed among spatial scales (Brown–Forsythe test, *P *<* *0.001). Specifically, there was a dramatic contraction of variance in hermaphrodite frequency with increasing spatial scales (Fig. 3). The phenotypic covariance among social partners (*C* ^*ij*′^) also decreased markedly with increasing spatial scale (Fig. 4).

Finally, the correlations between the local hermaphrodite frequencies an individual experienced decreased as the size of the spatial scales diverged (Table 3). The strongest correlations were between the closest spatial scales, such that increasing off‐diagonals of the correlation matrix show decreasing values. Thus, the hermaphrodite frequency that an individual experiences at one spatial scale becomes largely uncorrelated with hermaphrodite frequency experienced at spatial scales that differ by a meter or more. Taken together, these results all suggest that the hermaphrodite frequency that an individual experiences is strongly affected by the scale at which social interactions occur. This pattern may reflect differences in sampling variance of the sex ratio of the population, but would nonetheless be biologically relevant in the context of pollination or other interindividual interactions.

**Table 3:** Correlation matrix of local hermaphrodite frequencies across spatial scales. Rows and columns are the radius of the spatial scale, and values within are the correlation between the hermaphrodite frequencies individuals experience across scales indicated by the column and row pair. Cells are shaded to highlight the strength of the correlation; darker shading represents a higher correlation

	0.5	1.0	1.5	2.0	2.5	3.0	3.5	4.0	4.5	5.0	5.5	6.0
0.5	1.00	0.70	0.56	0.49	0.39	0.31	0.23	0.18	0.12	0.12	0.12	0.08
1.0		1.00	0.78	0.65	0.55	0.45	0.38	0.32	0.24	0.22	0.21	0.17
1.5			1.00	0.83	0.69	0.58	0.48	0.42	0.33	0.29	0.26	0.23
2.0				1.00	0.86	0.75	0.64	0.55	0.43	0.38	0.33	0.29
2.5					1.00	0.88	0.78	0.68	0.56	0.50	0.46	0.40
3.0						1.00	0.90	0.81	0.70	0.63	0.56	0.49
3.5							1.00	0.91	0.82	0.73	0.67	0.58
4.0								1.00	0.93	0.85	0.77	0.69
4.5									1.00	0.94	0.87	0.80
5.0										1.00	0.94	0.86
5.5											1.00	0.94
6.0												1.00

## Discussion

Social selection is generally studied as a population‐level phenomenon. The questions most often asked with regard to social selection are questions of how population‐level characteristics affect the fitness consequences of individual phenotypes. However, when a population exhibits fine‐scale phenotypic structure, there is potential for heterogeneity in local interactions across the population, which can result in different fitness consequences among individuals. Our results demonstrate that the distribution of the sexes in wild populations of *Silene vulgaris* creates the potential for dramatic differences in the social context experienced among individuals, depending on the scale of social interactions. We found that the patterns of phenotypic covariance of sex were greatest at the smallest spatial scales and disappeared at larger scales. These phenotypic covariances will have the greatest potential fitness effects on females, because a positive phenotypic covariance should exacerbate pollen limitation in female plants but not in hermaphrodite plants. This finding is particularly interesting in the context of vector‐pollinated plant populations, because the scale of pollinator movement is often among nearest neighbors (Waser 1982). This heterogeneity in the social environment at a fine scale, and the resulting potential for heterogeneity in selection differentials among individuals in a population, demonstrates that an understanding of the evolution of interacting phenotypes requires understanding the scale on which those interactions occur.

If we make some assumptions about differences in fitness between hermaphrodites and females in these populations, we can use the model of social selection to determine how the patterns of phenotypic covariance we found might impact sex ratio evolution. Although we did not measure fitness in these populations, we can make reasonable estimates based on past work. Previous work suggests females experience a twofold fitness advantage over hermaphrodites in fruit set or seed set (Olson et al. 2006; Dufay and Billard 2012). We can use this fitness measure and consider sex as our trait of interest to calculate the natural selection differential for these populations (see Appendix S1 for details of this calculation). The resulting natural selection differential on sex is −0.35, representing the inherent advantage females have through higher seed production. In the absence of other influences from the social environment, we would expect to see an increase in females if sex is at least partially heritable. However, when pollen moves among neighbors within a 1.5 m or smaller radius, the scales over which we found the largest values of *C* ^*ij*′^, a moderate‐to‐high strength of social selection will ameliorate these fitness differences between the sexes. Indeed, the social selection differential would completely counteract the natural selection differential against hermaphrodites when *β*_*S*_ ≥ 1.66, such that there would be no difference in fitness between the sexes, despite the strong direct female advantage. Conversely, if pollen moves among plants at scales greater than 3.0 m, the strength of social selection would need to be 10× greater to overcome female advantage, a magnitude that is unlikely to occur.

Previous studies of *S. vulgaris* suggest that the fitness effects of local sex ratios can be substantial. Olson et al. (2005) demonstrated that variance in the sex ratio among wild populations explained differences in relative fitness between the sexes. The relative fitness of females declined by 15% in wild populations because females suffered (and hermaphrodites benefited) from experiencing a locally high frequency of their own sex. Additionally, Olson et al. (2006) surveyed sex ratio at a fine scale in two wild populations of *S. vulgaris* and found a negative correlation between fruit set and local female frequency; the relative fitness of females was 19% lower than hermaphrodites due to the fine‐scale spatial structure of the sexes within populations. Our results suggest that this type of social selection has the opportunity to occur simultaneously at several spatial scales within populations. We found that hermaphrodites and females will experience sex ratios biased toward their own sex, provided mating occurs over the scale of a few meters within populations. Thus, the population‐level fitness effects that have previously been demonstrated also are likely to occur among individuals within the population.

In *S. vulgaris*, pollination is driven both by diurnal and nocturnal insects (Marsden‐Jones and Turril 1957). Stone (2013) demonstrated that in *S. vulgaris* hermaphrodites were visited more often and for longer periods of time by diurnal insects, while nocturnal insects showed a less pronounced preference for hermaphrodites. Taken together, these results have several important implications. Moths have been shown to travel greater distances than bees or flies during pollination (Barthelmess et al. 2006). Whatever aspects of phenology (sexually dimorphic or otherwise) that affect which plants pollinators visit and what they do afterward have the potential to greatly impact the fitness of individuals, given the difference in dispersal behavior among groups of pollinators. If pollinators have consistent preferences for plants based on the phenotype of individuals or the phenotypes of aggregations of individuals, those preferences may accelerate or constrain the effects of social selection.

Past experiments investigating the scale dependence of the fitness effects of social and ecological context in other plant species have revealed mixed results. Barrett and Thomson (1982) found no effect of sex ratio or density on fruit set in *Aralia nudicaulis* up to a distance of 40 meters, which they interpreted to reflect extremely long distance pollen dispersal. Aizen (1997) found that in *Alstroemeria aurea* neither density nor sex ratio explained large amount of variation in pollen receipt among individuals. However, there was a strong effect of sex ratio on seed output, suggesting that pollination effectiveness is impacted by social context. Conversely, there are many examples of species in which there is a direct relationship between fruit and/or seed set and the density of compatible mates in both wind (Stehlik et al. 2008)‐ and vector‐pollinated plants (House 1992), and these patterns often vary across spatial scales (Lin et al. 2015). Bartkowska and Johnston (2014) recently demonstrated the scale dependence of sex ratio and density in *Lobelia cardinalis*. Plants experienced negative fitness effects based on the phenotypes of neighbors within 10 centimeters, which the authors interpret as competition for resources, while plants experienced positive fitness effects due to the phenotypes of neighbors at scales greater than 10 centimeters, which they interpret as evidence for facilitation of pollinator visits. These examples highlight how the fitness consequences of social phenotypes can depend on the scale over which interactions occur.

The environmental and genetic factors that drive the observed patterns of spatial structure may alter predictions of any short‐ or long‐term responses to social selection. The environment – specifically light and moisture conditions – has been shown to affect sex expression and sex ratio in *S. vulgaris* (Dykstra et al. 2009) and *Geranium maculatum* (Van Etten and Chang 2009). Inbreeding also has been shown to significantly affect sex expression in *S. vulgaris –* the sex ratios of more inbred plants became increasingly female‐biased in a greenhouse crossing study (Glaettli and Goudet 2006). Additionally, there is evidence of postzygotic barriers to gene flow among plants that are spatially proximate and/or highly related (Glaettli et al. 2006). Taylor et al. (2001) demonstrated that the frequency of hermaphrodites of offspring is heritable, suggesting that the patterns of sex structuring may be maintained across generations. Any or all of these factors may determine the spatial distribution of plants in the wild or the timing of reproduction, which will determine the pool of potential mates that are available to individuals. Thus, any response to social selection will likely depend additionally on these environmental and genetic constraints.

The interactions that give rise to context‐dependent processes are complex and likely vary among individuals within populations. The scale over which social interactions occur is critical to understanding how social context can affect phenotypic evolution. Whether and how social interactions demonstrate scale dependence is an open question in plants and will vary with aspects of the mating system and ecology of the species. Social selection analysis presents a general empirical framework to investigate such questions and provides standardized metrics that are directly comparable. Although studies of social selection have mostly been conducted in animal species, the approach is equally valid and useful when applied to plant species, especially with respect to competition for resources or sunlight, herbivory, and pollination.

## Data Accessibility

Python and R scripts, GPS, and phenotypic data are deposited in Dryad: http://dx.doi.org/10.5061/dryad.c06n3.

## Conflict of Interest

None declared.

## Supporting information


**Appendix S1.** Supplemental methods.
