# Supplementary material for: Scale dependence of sex ratio in wild plant populations: implications for social selection
Source: Ecol Evol. 2016 Feb 3;6(5):1411–9. doi: 10.1002/ece3.1958 (PMC4739348; doi:10.1002/ece3.1958)
Supplement: Supplementary file 1 — Appendix S1. Supplemental methods. [file ECE3-6-1411-s001.docx]

Supplemental Methods

To estimate how the patterns of phenotypic covariance we measured might impact sex ratio evolution, we can use sex as the trait of interest (with hermaphrodites coded as 1 and females coded as 0) and estimate the natural and social selection differentials. We assigned values for fitness to each individual (1 for hermaphrodites, and 2 for females). In order to obtain standardized selection gradients we variance-standardized the index of sex, and obtained the values of relative fitness for each individual (0.73 for hermaphrodites, and 1.45 for females). We then regressed relative fitness on the standardized sex index, resulting in a value of $\beta_{N}=-0.35$. Because we are estimating a standardized selection gradient, the trait variance $P_{ii}$ = 1, and so the resulting natural selection differential is $P_{ii}\beta_{N}=-0.35$.

To illustrate how the scale of social interactions affects the fitness consequences of sex, we can estimate the magnitude of social selection that would be necessary to ameliorate this female advantage. For this species it is advantageous for both hermaphrodites and females to be surrounded by hermaphrodites, and so we expect that $\beta_{S}$ would be positive. At the 1.5 m scale we calculated a phenotypic correlation of $C^{ij^{'}}=0.21$, and so at that scale when the magnitude of social selection is $\beta_{S}=1.66$ the natural and social selection differentials cancel out, and there is no difference in net selection on sex despite a two-fold fitness advantage in females. At the 6.0 m scale $C^{ij^{'}}=0.025$, and so when pollinators move across very large scales in the population the strength of social selection would need to be 10 times greater to overcome female advantage.
